# Functional mechanism of miR-92b-3p in osteogenic differentiation of fibroblasts in patients with ankylosing spondylitis via the TOB1/BMP/Smad pathway

**DOI:** 10.1186/s13018-023-03850-1

**Published:** 2023-06-02

**Authors:** Liansong Lu, Shaohua Sun, Haojie Li, Yingzhi Xie

**Affiliations:** 1 Department of Spinal Surgery, Ningbo No.6 Hospital, 1059 East Zhongshan Road, Yinzhou District, Ningbo, 315040 Zhejiang China; 2 Department of Medical Image, Ningbo No.6 Hospital, 1059 East Zhongshan Road, Yinzhou District, Ningbo, 315040 Zhejiang China

**Keywords:** Ankylosing spondylitis, Fibroblasts, Osteogenic differentiation, miR-92b-3p, TOB1, BMP/Smad pathway

## Abstract

**Background:**

Ankylosing spondylitis (AS) is a chronic inflammatory arthritis. Upregulation of microRNA (miR)-92b-3p is associated with enhanced osteoblastic differentiation. The current study sought to investigate the functional mechanism of miR-92b-3p in osteogenic differentiation of AS fibroblasts.

**Methods:**

First, fibroblasts were isolated from AS and non-AS patients and cultured. Next, cell morphology was observed, cell proliferation was assessed and the vimentin expression pattern was determined. Alkaline phosphatase (ALP) activity and levels of osteogenic markers RUNX2, OPN, OSX, and COL I were additionally measured, followed by determination of miR-92b-3p and TOB1 levels. The binding site of miR-92b-3p and TOB1 was predicted, and their target relationship was validated. Lastly, miR-92b-3p inhibitor, si-TOB1, and the BMP/Smad signaling pathway inhibitor LDN193189 were delivered into AS fibroblasts to evaluate the osteogenic differentiation of AS fibroblasts and the activation of the BMP/Smad pathway.

**Results:**

miR-92b-3p was highly expressed in AS fibroblasts. AS fibroblasts showed enhanced osteogenic differentiation and proliferation, while inhibition of miR-92b-3p suppressed osteogenic differentiation and proliferation of AS fibroblasts. miR-92b-3p targeted TOB1, and TOB1 was poorly expressed in AS fibroblasts. The concurrent downregulation of TOB1 and inhibition of miR-92b-3p elevated the levels of RUNX2, OPN, OSX, and COL I and ALP activity and further enhanced the proliferation of AS fibroblasts. The BMP/Smad pathway was activated in AS fibroblasts. Silencing miR-92b-3p could inhibit the activation of the BMP/Smad pathway by upregulating TOB1. Inhibition of the BMP/Smad pathway reduced the number of calcified nodules and hindered the osteogenic differentiation and proliferation of AS fibroblasts.

**Conclusion:**

Our findings highlighted that silencing miR-92b-3p inhibited the osteogenic differentiation and proliferation of AS fibroblasts by upregulation of TOB1 and inhibition of the BMP/Smad pathway.

**Graphical abstract:**

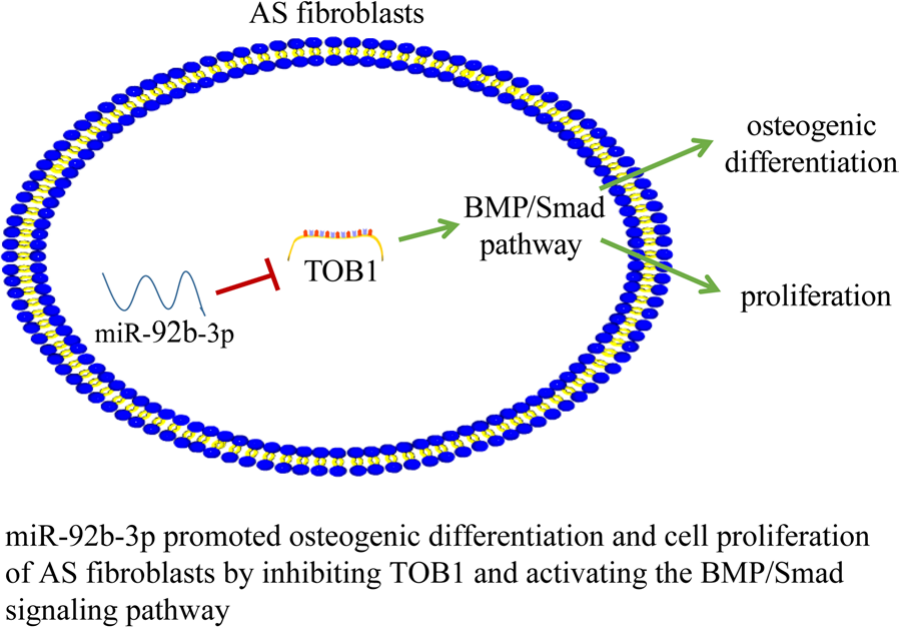

**Supplementary Information:**

The online version contains supplementary material available at 10.1186/s13018-023-03850-1.

## Introduction

Ankylosing spondylitis (AS) is a chronic inflammatory rheumatic disease characterized by progressive back pain, stiffness, and limited spinal mobility [1]. After chronic inflammation, the newly formed bones are highly susceptible to advancing ossification and ankylosis of the spine and sacroiliac joint [2]. Several factors such as genetic and environmental factors have been regarded as the primary causes of AS [3]. The predominant incidence of AS is among young adults aged between 20 and 30 years, and most patients are initially presented with radical back pain and stiffness, which is further exacerbated by potential risks for osteoporosis, spinal compression fractures, iritis, and cardiovascular diseases [4]. Fundamentally, fibroblasts are the principal cell components of ossified connective tissues around the vertebral column and joints in AS [5]. The reinforced differentiation of osteoblasts is the basis of new bone formation [6]. An existing study has elicited the potential of fibroblasts to differentiate into osteoblasts and express osteogenic marker genes [7]. Therefore, a close association between fibroblasts and AS pathogenesis is speculated, and a comprehensive understanding of the osteogenic differentiation of fibroblasts is of vital importance for the development of new therapeutic strategies for AS (Additional files 1, 2, 3).

Accumulating evidence has elicited the vital functionality of non-coding small molecular RNAs in several biological processes including cell proliferation and differentiation, immune response, and aging [8–10]. Essentially, microRNAs (miRNAs) are a class of small non-coding RNAs [11] that by complex interactions with numerous target genes can serve as vital modulators of gene regulation in osteoarthritis and maintenance of homeostasis pathways [12]. Additionally, the functionality of miRNAs in AS pathology has been extensively studied [13]. For instance, miR-214-3p can radically inhibit AS fibroblast osteogenesis via regulation of bone morphogenetic protein 2 (BMP2) and the BMP/TGF-β axis [14], while miR-204-5p can suppress the osteogenic differentiation of AS fibroblasts by modulating the Notch2 signaling pathway [15]. Notably, miR-92b and miR-92b-5p can facilitate the osteogenic differentiation of mesenchymal stem cells [16, 17]. Unfortunately, miR-92b-3p has been rarely studied. What’s more, miR-92b-3p exhibits high expression levels in breast cancer and gastric cancer [18, 19]. Therefore, we hypothesized that miR-92b-3p might potentiate the osteogenic differentiation of AS fibroblasts.

Transducer of ERBB2-1 (TOB1), a negative regulator of the BMP/Smad signaling pathway, has been reported to suppress bone formation in adult animals without altering bone resorption [20]. Moreover, TOB1 plays a key role in miR-26a-mediated regulation of osteogenesis under osteoporotic conditions [21]. Therefore, we speculated whether miR-92b-3p could influence osteogenic differentiation of AS fibroblasts by targeting TOB1. MMP-2 gene silencing has been demonstrated to reduce the degree of osteogenic differentiation of AS fibroblasts by inhibiting activation of the BMP/Smad pathway [22]. Hence, we speculated that miR-92b-3p functioned in AS by modulating osteogenic differentiation of AS fibroblasts via the TOB1-mediated BMP/Smad pathway. The current study sought to investigate the underlying mechanism of miR-92b-3p in regulating the osteogenic differentiation of AS fibroblasts to achieve a comprehensive understanding of the pathogenic mechanism of AS.

## Material and methods

### Ethics statement

The experiments involving human specimens were conducted in strict accordance with the guidelines of the institutional review board and with the approval of the Ethics Committee of Ningbo No.6 Hospital (Approval Number: L2022102). Informed consent for biological material collection and research was provided by the patients and their families.

### Study subjects

The specimens of hip joint capsule tissues were isolated from patients hospitalized in the Ningbo No.6 Hospital and undergoing surgical intervention from January 2019 to January 2021. The experimental group comprised 5 AS patients (3 males, 2 females; aged about 33.8 years), while 5 patients (4 males, 1 female; aged about 34.2 years) with spinal injury and no other immune diseases such as rheumatism were assigned to the control group. The aforementioned patients complied with the recommendations of the New York Standard of the American Rheumatism Association revised in 1984.

### Cell isolation, culture, and identification

Fibroblasts were isolated from the hip joint capsule tissues of AS patients and controls. All experimental procedures were performed on a clean and sterile bench. Briefly, the tissue specimens were isolated from patients on an operating table and immediately placed in a sterile D-Hanks solution (Thermo Fisher Scientific, Waltham, MA, USA). The residual blood and attached tissues were eliminated by 2–3 consecutive rinses. Next, the tissues were incised into small pieces and incubated in Dulbecco's modified Eagle's medium (DMEM; Gibco, Grand Island, NY, USA) supplemented with a combination of 10% fetal bovine serum (FBS), 100 U/mL penicillin and 100 μg/mL streptomycin (Biolight, Shanghai, China) at 37 °C for 5 h with 5% CO_2_ to facilitate complete adherence of the tissue blocks to the wall. The tissues were cultured for 3–4 days to produce cell climbing. Upon 80% cell coverage of the bottom of the bottle, the cells were harvested and rinsed with sterile D-Hanks solution 3 times, detached using 0.25% trypsin (TransDetect, Beijing, China) and then, subcultured. The cells were cultured with 5% CO_2_ at 37 °C. Fibroblasts at the 3^rd^ passage were stained with hematoxylin and eosin (HE) in strict accordance with the provided instructions of the HE staining kit (Beyotime, Shanghai, China) for morphological observation. Meanwhile, the expression pattern of vimentin (the specific protein in fibroblasts) was measured by immunocytochemistry.

### Immunocytochemistry

The clean coverslips were placed in a culture medium, supplemented with a suspension of fibroblasts at the 3rd passage, and cultured for 24–48 h. The medium was removed after cells covered the coverslips. The coverslips were rinsed using phosphate buffer saline (PBS), fixed with 4% paraformaldehyde for 15 min, and incubated with 0.3% Triton X-100 solution for 20 min. After a rinse with PBS, a fibroblast blockade was conducted with 1% FBS for 30 min, incubated with monoclonal mouse anti-vimentin (at a dilution ratio of 1:100, ab8978, Abcam, Cambridge, MA, USA) for 1 h, and then, incubated with the goat anti-mouse IgG (at a dilution ratio of 1:2000, ab205719, Abcam) for 30 min. Next, the fibroblasts were stained with 2, 4-diaminobutyric acid and then, counterstained with hematoxylin prior to microscopic observation (TS100, Nikon, Kanagawa, Japan).

### Cell treatment and grouping

The experimental fibroblasts were classified into 8 groups: (1) normal group (fibroblasts isolated from hip joint capsule tissues of patients in the control group), (2) AS group (fibroblasts isolated from hip joint capsule tissues of AS patients), (3) AS + miR-inhi group (AS fibroblasts transfected with miR-92b-3p inhibitor), (4) AS + miR-inhi-NC group (AS fibroblasts transfected with the negative control of miR-92b-3p inhibitor), (5) AS + miR-inhi + si-TOB1 group (AS fibroblasts transfected with miR-92b-3p inhibitor and si-TOB1), (6) AS + miR-inhi + si-NC group (AS fibroblasts transfected with miR-92b-3p inhibitor and si-NC), (7) AS + miR-inhi + si-TOB1 + LDN193189 group [AS fibroblasts cultured in osteogenic induction medium supplemented with 100 nM/mL BMP/Smad pathway inhibitor LDN193189 (SF7912-5 mg, Beyotime) for 48 h after transfection with miR-92b-3p inhibitor and si-TOB1], and (8) AS + miR-inhi + si-TOB1 + PBS group (AS fibroblasts cultured with an equal amount of PBS for 48 h after transfection with miR-92b-3p inhibitor and si-TOB1).

### Cell transfection

The fibroblasts at the 3rd passage with 80% confluence were transfected with the miR-92b-3p inhibitor (5′-GGAGGCCGGGACGAGUCAAUA-3′) and its negative control inhibitor-NC (GenePharma, Shanghai, China). Next, si-TOB1 (5′-GCUGUAAGCCCUACCUUCATT UGAAGGUAGGGCUUACAGCTT-3′) and its negative control si-NC (GenePharma) were introduced into fibroblasts after transfection with miR-92b-3p inhibitor. Cell transfection was performed in strict accordance with the provided instructions of the Lipofectamine™2000 transfection kit (Invitrogen, Thermo Fisher Scientific) at the final concentration of 50 nM. After 48 h of transfection, fibroblasts were isolated for subsequent experimentation. The transfection efficiency was determined by means of reverse transcription-quantitative polymerase chain reaction (RT-qPCR).

### Alizarin red staining (ARS)

When fibroblasts at the 3rd passage reached 80% confluence, osteogenic differentiation was induced by adding 0.1 μmol/L dexamethasone + 10 mmol/L β-glycerophosphate + 50 μmol/L ascorbic acid [14]. Subsequent experiments were conducted using the osteogenic induction medium. On day 21 after osteogenic induction, the fibroblasts were fixed using 4% paraformaldehyde and stained with 1% ARS (pH 4.3) at 23 ± 2 °C for 15 min. The experimental findings were documented. Next, 10% ammonium hydroxide (Sigma-Aldrich, St Louis, MO, USA) solution was added for dilution of the calcium deposits and the absorbance value at a wavelength of 405 nm was measured using a spectrophotometer (Biotek, Winooski, VT, USA) for quantitative analysis. ARS quantification was estimated based on a standard curve [23].

### Alkaline phosphatase (ALP) activity detection

The activity of ALP was detected using the ALP qualitative detection kit (Nanjing Jiancheng Bioengineering Institute, Nanjing, Jiangsu, China). The fibroblasts at the 3rd passage were seeded in 12-well plates equipped with coverslips at a diameter of 10 mm. The coverslips were removed after 21 days of induction, rinsed twice using PBS, and fixed with 4% paraformaldehyde for 8 min. Next, fibroblasts were rinsed twice using PBS, supplemented with the ALP substrate for 30 min at 37 °C, rinsed, sealed, and observed under an inverted microscope, with the experimental findings documented.

The ALP activity was detected using the provided ALP kit (Nanjing Jiancheng Bioengineering Institute). The fibroblasts at the 3rd passage were isolated and lysed on the 7th, 14th, and 21st day of osteogenic induction, supplemented with 100 μL of reaction substrate for 15 min at 37 °C. The reaction was terminated by the addition of a termination solution. The absorbance value at a wavelength of 405 nm was detected using a microplate reader (Bio-Rad 680, Bio-Rad, Hercules, CA, USA), and the ALP activity was calculated.

### RT-qPCR

The total RNA content was extracted from fibroblasts using the TRIzol kit (R&D Systems Inc., Minneapolis, MN, USA). The complementary DNA (cDNA) was synthesized using the TaqMan MicroRNA assay (PE Applied Biosystems, Foster City, CA, USA) and PrimeScript™ RT reagent kits (all from Takara Bio, Tokyo, Japan). According to the total RNA concentration of 516.4 ng/μL, reverse transcription was performed using 2 μL of total RNA at 37 °C for 15 min followed by 85 °C for 5 s. RT-qPCR was conducted on the Applied Biosystems 7800 Sequence Detection System. The obtained cDNA was subjected to PCR amplification using the following thermocycling conditions: denaturation at 92 °C for 10 s, annealing at 55 °C for 20 s, and extension at 68 °C for 20 s (40 cycles). The relative expression levels of miR-92b-3p, runt-related transcription factor 2 (Runx2), osteopontin (OPN), osterix (OSX), and collagen type I (COL I) were quantified using the SYBR Green PCR kit (Takara Bio), and U6 or GAPDH was used as the internal reference. The threshold cycle (CT) data were determined under the default threshold settings. All RT-qPCR procedures were conducted three times independently. The values of average CT and standard deviation (SD) were estimated. The relative gene expression was calculated using the 2^−ΔΔCt^ method. Primer sequences are provided in Table 1.

**Table 1:** Primer sequences in RT-qPCR

Name	F (5′-3′)	R (5′-3′)
miR-92b-3p	CCGGCCCCGGCCCCCC	AGTGCAGGGTCCGAGGTATT
RUNX2	CAAGGACAGAGTCAGATTAC	GTGGTAGAGTGGATGGAC
OPN	CTCCATTGACTCGAACGACTC	CAGGTCTGCGAAACTTCTTAGAT
OSX	TGCTTGAGGAGGAAGTTC	CTTTGCCCAGAGTTGTTG
COL I	GAGGGCCAAGACGAAGACATC	CAGATCACGTCATCGCACAAC
GAPDH	CCATCTTCCAGGAGCGAGATC	GCCTTCTCCATGGTGGTGAA
U6	CTCGCTTCGGCAGCACA	AACGCTTCACGAATTTGCGT

RT-qPCR, reverse transcription-quantitative polymerase chain reaction; miR, microRNA; RUNX2, runt-related transcription factor 2; OPN, osteopontin; OSX, osterix; COL I, collagen I; GAPDH, glyceraldehyde-3-phosphate dehydrogenase; F, forward; R, reverse

### Western blot

Total protein of fibroblasts was extracted as per the provided instructions of the Protein Extraction kit (Beyotime), and its concentration was measured using a bicinchoninic acid Protein Assay kit (Beyotime). Then, 50 μg of protein sample was loaded onto 12% sodium dodecyl sulfate–polyacrylamide gel electrophoresis for 90 min of electrophoresis on ice. The separated protein was transferred onto polyvinylidene fluoride membranes, which were subsequently blocked for 1 h in Tween-20 (TBST, 20 nM Tris, 137 mM NaC1, 0.1% Tween-20) Tris buffer containing 5% non-fat milk. After that, the membranes were incubated with rabbit monoclonal anti-TOB1 (A-AP8571a, at a dilution ratio of 1:1000, Amyjet Scientific, Wuhan, Hubei, China), rabbit monoclonal anti-BMP-2 (ab214821, at a dilution ratio of 1:1000, Abcam), rabbit monoclonal anti-Smad1 (ab126761, at a dilution ratio of 1:1000, Abcam), and rabbit polyclonal p-Smad1/5/9 (ab80255, at a dilution ratio of 1:1000, Abcam) at 4 °C overnight. After a rinse, the membranes were incubated with HRP-labeled secondary antibody goat anti-rabbit IgG (ab205718, at a dilution ratio of 1:2000, Abcam) for 2 h. The protein band was developed using an ECL kit (Seyotin, Guangzhou, China). Gray value analysis was conducted using ImageJ software (National Institutes of Health, Bethesda, MD, USA). GAPDH (ab9485, at a dilution ratio of 1:2500, Abcam) and β-actin (ab8227, at a dilution ratio of 1:1000, Abcam) were regarded as internal controls.

### Cell counting kit 8 (CCK-8)

Cell proliferation was assessed using the CCK-8 kit (Sigma-Aldrich). The cells in different groups were seeded in 96-well plates at the density of 6 × 10^3^ cells/well. Subsequently, 10 µL of CCK-8 solution (Sigma-Aldrich) was supplemented at the time points of 24, 48, and 72 h of culture to incubate with cells for 2 h. Ultimately, the absorbance value of each well at a wavelength of 450 nm was detected.

### Dual-luciferase reporter assay

TOB1 sequences containing the miR-92b-3p binding sites and its mutant sequences were amplified and inserted into the pmiR-GLO luciferase vector (Promega, Madison, WI, USA, 250 ng) containing the firefly luciferase, and pTK-Green Renilla Luc (Thermo Fisher Scientific, 50 ng) by PCR. The wild-type plasmid of luciferase reporter gene (TOB1-WT) (F: 5′-CCGGAGCTCGCCACCAUGGATGAAGTTTAAAGATTTTTGCTATATATTATGGAAGAAAAATG-3′; R: 5′-CGTCTAGATAATTTTAAAGAATTTTAATACAAACTTAATATAAACTATTTCAGTCCCT-3'), and corresponding mutant plasmid (TOB1-MUT) (F: 5′-CCGGAGCTCGCCACCAUGGATTAATTTTGTACCTATATTCACGTTAACTTGAAAAAAACGGTAT-3′; R: 5′-CGTCTAGAATACCGTTTTTTTCAAGTTAACGTGAATATAGGTACAAAATTAAT-3′) were constructed by GenePharma. HEK293 cells (ATCC, Manassas, VA, USA) were cultured in DMEM supplemented with 10% FBS at 37 °C with 5% CO_2_. The HEK293 cells were seeded in 6-well plates (1 × 10^5^ cells/well) and subsequently incubated for 24 h. Then, the HEK293 cells were co-transfected with the constructed luciferase vectors containing TOB1-WT or TOB1-MUT, and miR-mimic-NC or miR-92b-3p-mimic (5'-UAUUGCACUCGUCCCGGCCUCCAGGCCGGGACAGUGCAAUAUU-3′) (GeneChem) (miRNA-mimic, 30 nM) using Lipofectamine 2000 (Invitrogen, Thermo Fisher Scientific). After 48-h transfection, the luciferase activity was detected using the Dual-luciferase reporter assay system (E1960, Promega). The relative luciferase activity of each sample was estimated as the ratio of firefly luciferase activity to Renilla luciferase activity.

### Statistical analysis

The experimental data were analyzed and plotted using SPSS 21.0 (IBM Corp., Armonk, NY, USA) and GraphPad Prism 8.01 (GraphPad Software Inc., San Diego, CA, USA). Pairwise comparisons were analyzed using the *t* test, and comparisons among multiple groups were analyzed using one-way analysis of variance (ANOVA), followed by Tukey’s multiple comparisons test. In all statistical references, a value of *p* < 0.05 was indicative of statistical significance, and a value of *p* < 0.01 was regarded as extremely statistically significant.

## Results

### miR-92b-3p was highly expressed in AS fibroblasts

**Fig. 1:**
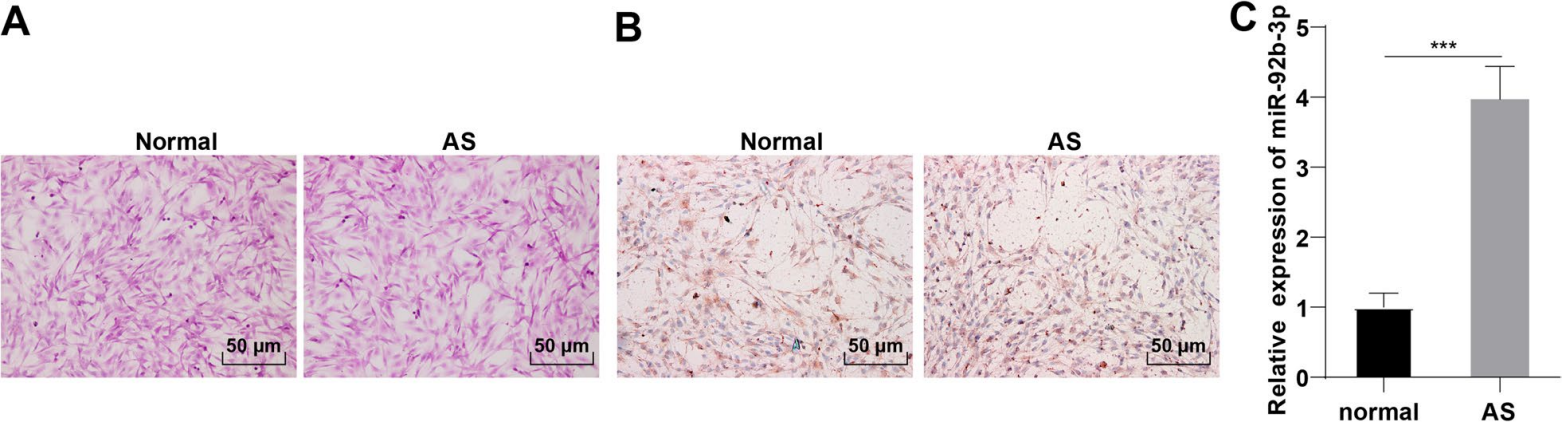
miR-92b-3p was highly expressed in AS fibroblasts. Fibroblasts were isolated from hip joint capsule tissues of AS patients and controls and cultured separately. **A**: cell morphology observed by HE staining; **B**: positive vimentin expression detected by immunocytochemistry; **C**: expression levels of miR-92b-3p measured by RT-qPCR. Cell experiment was repeated 3 times. Data were presented as mean ± standard deviation. Pairwise comparisons were analyzed using independent sample *t* test. ***p < 0.001

In order to investigate whether miR-92b-3p is implicated in osteogenic differentiation of AS fibroblasts, fibroblasts were initially isolated from the hip joint capsule tissues of AS patients and patients with spinal injury and no prior history of other immune diseases such as rheumatism. HE staining presented no certain morphological differences between the AS fibroblasts and normal fibroblasts, which were in long fusiform shape with regular oval nuclei (Fig. 1A). Furthermore, vimentin expression was detected by immunocytochemistry, which revealed positive vimentin expression in AS fibroblasts and normal fibroblasts, with stronger vimentin expression in AS fibroblasts than that in normal fibroblasts (Fig. 1B), thus signifying that all cultured fibroblasts were mesodermal fibroblasts in origin. Subsequently, RT-qPCR revealed elevated miR-92b-3p expression levels in AS fibroblasts relative to those in normal fibroblasts (*p* < 0.001, Fig. 1C).

### Silencing miR-92b-3p inhibited AS fibroblast proliferation and osteogenic differentiation

**Fig. 2:**
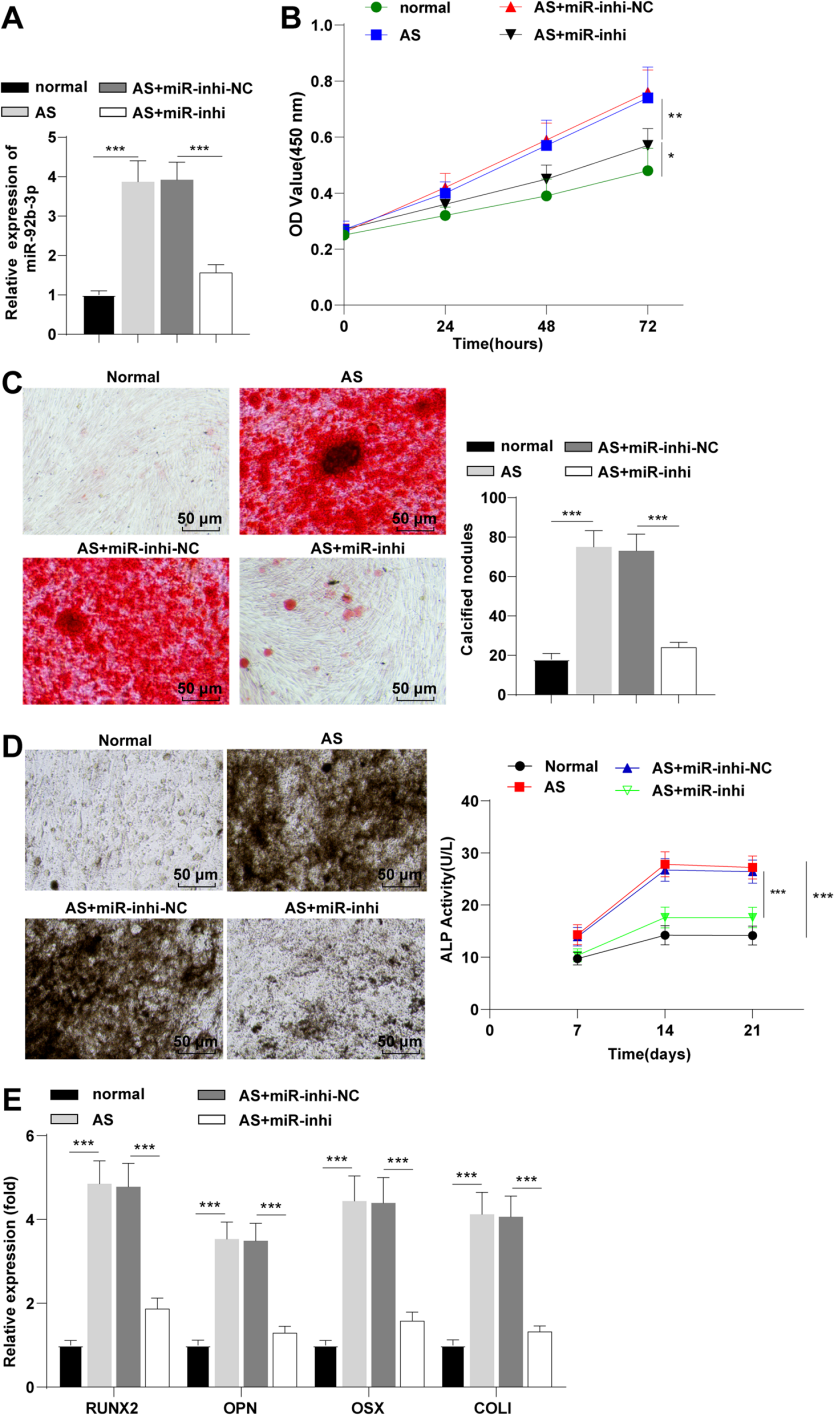
Silencing miR-92b-3p inhibited proliferation and osteogenic differentiation of AS fibroblasts. miR-92b-3p inhibitor and miR-92b-3p inhibitor-NC were introduced into AS fibroblasts. **A**: expression levels of miR-92b-3p measured by RT-qPCR; **B**: cell proliferation evaluated by CCK-8; **C**: osteogenic differentiation and calcified nodule formation analyzed by ARS; **D**: ALP activity assessed by ALP qualitative and quantitative kit; **E**: mRNA expression levels of osteogenic markers RUNX2, OPN, OSX, and COL I determined by RT-qPCR. Cell experiment was conducted 3 times independently. Data were presented as mean ± standard deviation. Comparisons among multiple groups were analyzed using one-way ANOVA, followed by Tukey's multiple comparisons test. *p < 0.05, **p < 0.01, ***p < 0.001

To further verify the functions of miR-92b-3p in proliferation and osteogenic differentiation of AS fibroblasts, miR-92b-3p-inhibitor was subsequently delivered into AS fibroblasts in an attempt to silence miR-92b-3p. The results of RT-qPCR revealed reduced expression levels of miR-92b-3p in the AS + miR-inhi group relative to those in the AS + miR-inhi-NC group (*p* < 0.001, Fig. 2A), thus indicative of successful miR-92b-3p silencing by transfection. After 21 days of osteogenic induction, CCK-8 assay showed that AS fibroblasts had stronger proliferative ability than normal fibroblasts (*p* < 0.01), and inhibition of miR-92b-3p reduced AS fibroblast proliferation (*p* < 0.05, Fig. 2B). ARS showed intensified staining color with an elevated number of calcified nodules in AS fibroblasts relative to those in normal fibroblasts (*p* < 0.001), indicating that AS fibroblasts showed stronger osteogenic differentiation ability than normal fibroblasts, while miR-92b-3p silencing radically inhibited the osteogenic differentiation ability of AS fibroblasts (Fig. 2C). Meanwhile, AS fibroblasts demonstrated extensive staining and increased ALP activity relative to normal fibroblasts (*p* < 0.001), while the ALP staining color became lighter and ALP activity was reduced after silencing miR-92b-3p (*p* < 0.001, Fig. 2D). Moreover, the findings of RT-qPCR showed that the mRNA expression levels of RUNX2 (an essential regulator of osteogenic differentiation), OPN (fibroblast osteocalcin), OSX (osteoblast-specific transcription factor), and collagen I (COL I; fibroblast type 1 collagen) were all increased in AS fibroblasts (*p* < 0.001), and decreased after silencing miR-92b-3p (*p* < 0.001, Fig. 2E). Collectively, our findings revealed that silencing miR-92b-3p terminally inhibited the proliferation and osteogenic differentiation of AS fibroblasts.

### miR-92b-3p targeted TOB1

**Fig. 3:**
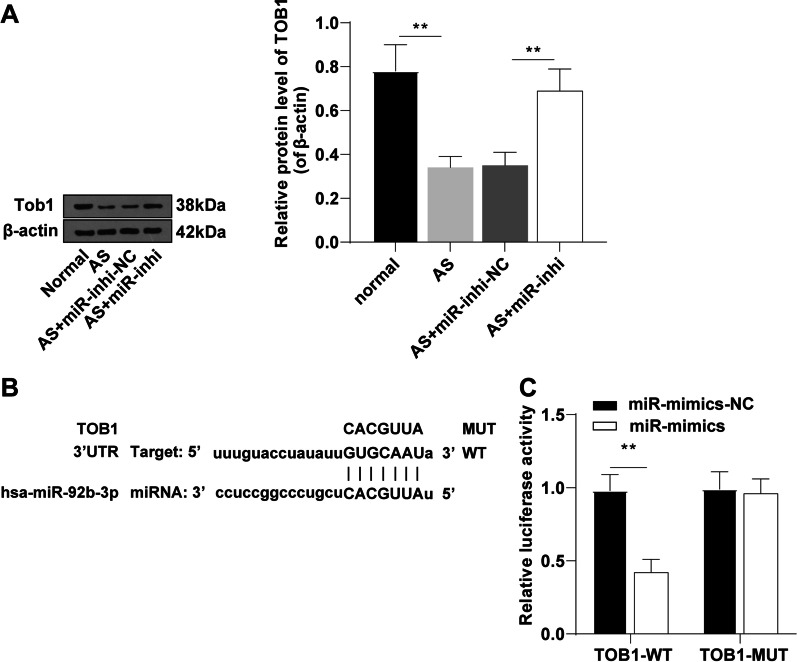
miR-92b-3p targeted TOB1. **A**: protein expression levels of TOB1 determined by Western blot; **B**: the binding site of miR-92b-3p and TOB1 predicted in the ENCORI database; **C**: target relationship between miR-92b-3p and TOB1 validated by dual-luciferase reporter assay. Cell experiment was conducted 3 times independently. Data were presented as mean ± standard deviation. Data in panel A were analyzed using one-way ANOVA, followed by Tukey's multiple comparisons test, and data in panel C were analyzed using the independent sample *t* test. **p < 0.01

Accumulating evidence has highlighted the participation of TOB1 in osteogenic differentiation [21, 24]. Western blot results revealed decreased expression levels of TOB1 in AS fibroblasts compared to those in normal fibroblasts (Fig. 3A). The binding site of miR-92b-3p and TOB1 was predicted using the ENCORI database (http://starbase.sysu.edu.cn/) (Fig. 3B). Therefore, we speculated the functionality of miR-92b-3p as a modulator of osteogenic differentiation of AS fibroblasts via TOB1. Dual-luciferase reporter assay presented reduced relative luciferase activity in HEK293 cells transfected with TOB1-WT and miR-92b-3p-mimic relative to that in the miR-mimic-NC group (*p* < 0.01, Fig. 3C), while no marked difference was evident in the luciferase activity in HEK293 cells transfected with TOB1 without the binding site of miR-92b-3p, which signified the target relationship between miR-92b-3p and TOB1.

To further validate the regulatory relationship between miR-92b-3p and TOB1, we analyzed the protein expression pattern of TOB1 in AS fibroblasts transfected with miR-92b-3p inhibitor. Western blot results showed elevated protein expression levels of TOB1 in AS fibroblasts after silencing miR-92b-3p relative to those in the AS group and AS + miR-inhi-NC group (*p* < 0.01, Fig. 3A), thus indicating that miR-92b-3p could negatively regulate TOB1 expression.

### TOB1 downregulation partially annulled the inhibitory effect of silencing miR-92b-3p on proliferation and osteogenic differentiation of AS fibroblasts

**Fig. 4:**
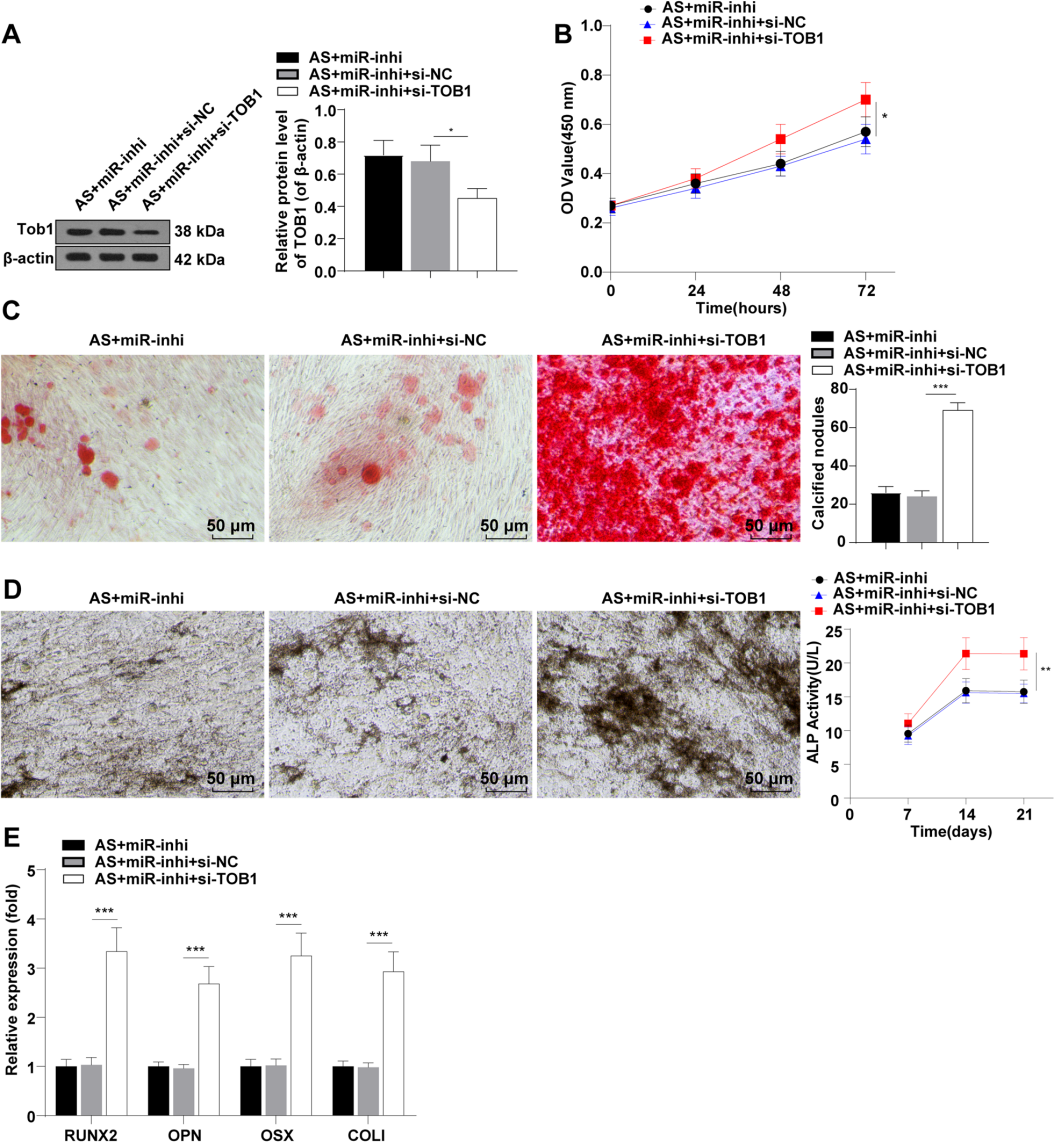
TOB1 downregulation partially annulled the inhibitory effect of silencing miR-92b-3p on the proliferation and osteogenic differentiation of AS fibroblasts. miR-inhi and si-TOB1 were co-introduced into AS fibroblasts. **A**: protein expression levels of TOB1 determined by Western blot; **B**: cell proliferation evaluated by CCK-8; **C**: calcified nodule formation analyzed by ARS; **D**: ALP activity assessed by ALP staining; E: mRNA expression levels of RUNX2, OPN, OSX, and COL I detected by RT-qPCR. Cell experiment was conducted 3 times independently. Data were presented as mean ± standard deviation. Comparisons among multiple groups were analyzed using one-way ANOVA, followed by Tukey's multiple comparisons test. *p < 0.05, **p < 0.01, ***p < 0.001

To further validate the roles of miR-92b-3p and TOB1 in the proliferation and osteogenic differentiation of AS fibroblasts, AS fibroblasts were subsequently transfected with si-TOB1 and miR-92b-3p inhibitor. Western blot revealed reduced protein expression levels of TOB1 in the AS + miR-inhi + si-TOB1 group relative to those in the AS + miR-inhi group (*p* < 0.05, Fig. 4A), which ascertained the inhibition of TOB1 expression in AS fibroblasts. On the 21st day of osteogenic induction, CCK-8 assay showed that TOB1 downregulation partly annulled the inhibitory action of silencing miR-92b-3p on AS fibroblast proliferation (*p* < 0.05, Fig. 4B). ARS showed an increased staining degree along with an elevated number of calcified nodules in the AS + miR-inhi + si-TOB1 group compared to those in the AS + miR-inhi group (*p* < 0.001, Fig. 4C). Additionally, increased ALP staining degree and ALP activity were observed in the AS + miR-inhi + si-TOB1 group relative to those in the AS + miR-inhi group (*p* < 0.01, Fig. 4D). RT-qPCR results revealed that TOB1 downregulation partially annulled the inhibitory effect of silencing miR-92b-3p on the levels of RUNX2, OPN, OSX and COL I (*p* < 0.001, Fig. 4E). Altogether, our findings demonstrated that TOB1 downregulation partially averted the inhibitory effect of silencing miR-92b-3p on the proliferation and osteogenic differentiation of AS fibroblasts.

### Silencing miR-92b-3p inhibited proliferation and osteogenic differentiation of AS fibroblasts via the TOB1/BMP/Smad axis

**Fig. 5:**
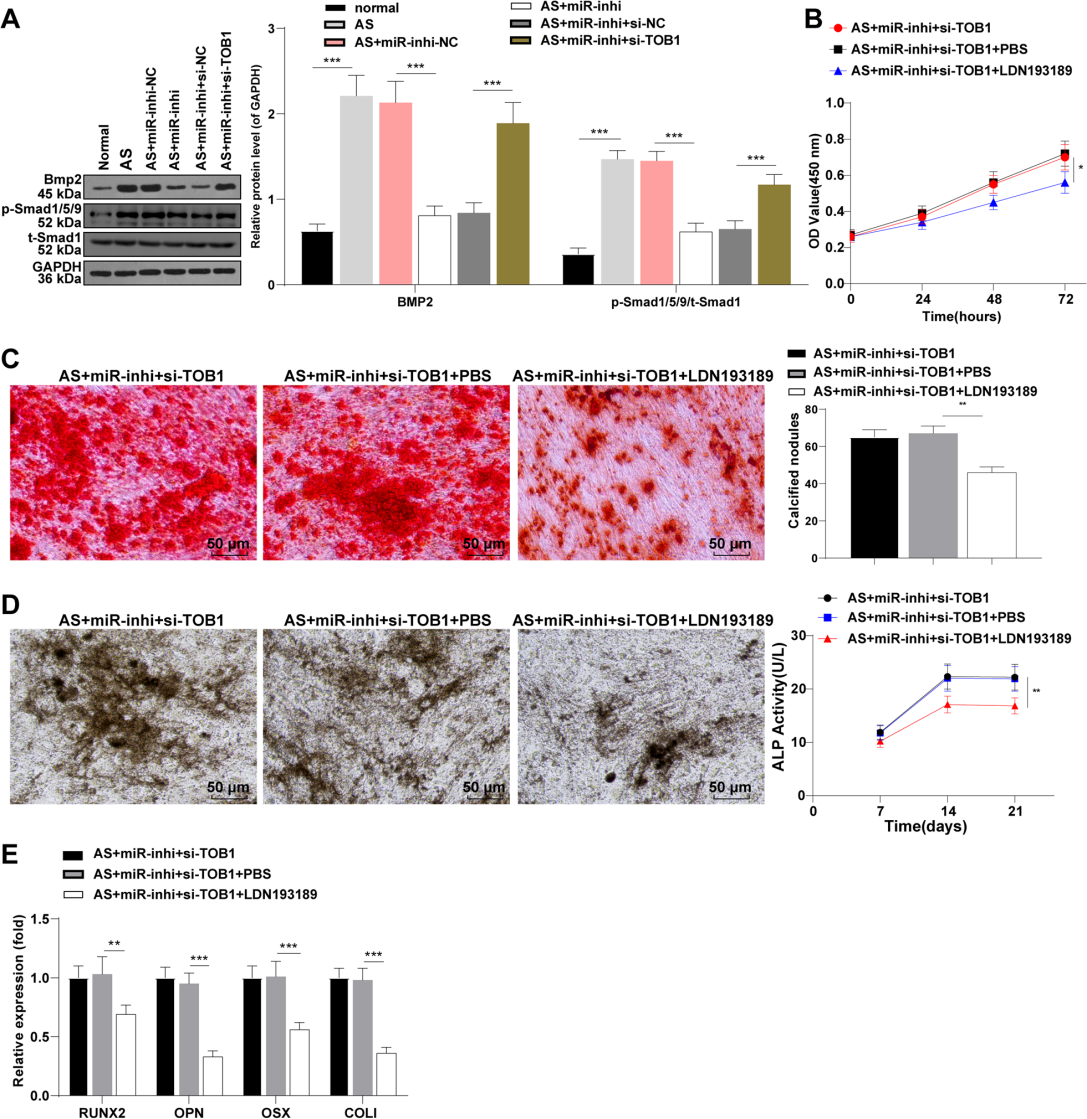
Silencing miR-92b-3p inhibited proliferation and osteogenic differentiation of AS fibroblasts via the TOB1/BMP/Smad axis. **A**: protein expression levels of BMP-2 and p-Smad1/5/9/t-Smad1 determined by Western blot; **B**: cell proliferation evaluated by CCK-8; **C**: calcified nodule formation analyzed by ARS; **D**: ALP activity assessed by ALP staining; **E**: mRNA expression levels of RUNX2, OPN, OSX and COL I determined by RT-qPCR. Cell experiment was conducted 3 times independently. Data were presented as mean ± standard deviation. Comparisons among multiple groups were analyzed using one-way ANOVA, followed by Tukey's multiple comparisons test. *p < 0.05, **p < 0.01, ***p < 0.001

TOB1 could potentially interact with Smad, a downstream regulator of BMP-2, to negatively modulate BMP-2 signaling during bone formation [25]. To further investigate whether miR-92b-3p could certainly manipulate the proliferation and osteogenic differentiation of AS fibroblasts via the TOB1-mediated BMP/Smad pathway, the expression levels of BMP-2 and p-Smad1/5/9/t-Smad1 (key proteins of BMP/Smad pathway) were determined by Western blot. The result exhibited elevated expression levels of BMP-2 and p-Smad1/5/9/t-Smad1 in AS fibroblasts compared to those in normal fibroblasts (all *p* < 0.001) and decreased expression levels after silencing miR-92b-3p (*p* < 0.001). After silencing miR-92b-3p and downregulating TOB1, the expression levels of BMP-2 and p-Smad1/5/9/t-Smad1 were elevated (*p* < 0.001, Fig. 5A). Briefly, our findings revealed that silencing miR-92b-3p suppressed activation of the BMP/Smad pathway by upregulating TOB1.

Furthermore, we verified the function of miR-92b-3p on the proliferation and osteogenic differentiation of AS fibroblasts via inhibition of the TOB1/BMP/Smad axis. The BMP/Smad pathway inhibitor LDN193189 was supplemented into the AS fibroblasts concurrently transfected with miR-92b-3p inhibitor and si-TOB1. Relative to the AS + miR-inhi + si-TOB1 + PBS group, the AS + miR-inhi + si-TOB1 + LDN193189 group exhibited a significant reduction in cell proliferation (*p* < 0.05, Fig. 5B). Additionally, a decrease in ARS and ALP staining degree, a reduction in the number of calcified nodules (*p* < 0.01), reduced ALP activity (*p* < 0.01), and downregulated mRNA expression levels of RUNX2 (*p* < 0.01), OPN (*p* < 0.001), OSX (*p* < 0.001), and COL I (*p* < 0.001) were observed (Fig. 5C–E). The aforementioned results suggested that the inhibition of the BMP/Smad pathway partially nullified the promotional action of TOB1 downregulation on AS fibroblast proliferation and osteogenic differentiation. Conjointly, our results elicited that miR-92b-3p modulated the proliferation and osteogenic differentiation of AS fibroblasts via blockade of the TOB1/BMP/Smad pathway.

## Discussion

AS is a prevalent form of spondyloarthropathies that can result in a progressive loss of joint function and disability [26]. AS pathogenesis is associated with miRNA-mediated immune cell functions such as cytokine response and T-cell survival [27]. Notably, the functionality of miR-92b-3p has been identified in the progression of osteoporosis [28]. We herein investigated the potential function of miR-92b-3p on osteogenic differentiation of AS fibroblasts via the TOB1/BMP/Smad pathway.

An existing study has demonstrated the positive roles of miR-92b and miR-92b-5p in the osteogenic differentiation of mesenchymal stem cells [16, 17]. Essentially, vimentin has been identified as a potential marker of fibroblasts [29]. Our results presented positive vimentin expression in AS fibroblasts and normal fibroblasts. Additionally, an elevated expression of miR-92b-3p was evident in the AS fibroblasts relative to normal fibroblasts, which was consistent with the finding of a previous study [30]. ALP activity and calcification have been classified as vital phenotypic markers of osteogenic differentiation [31]. Our result revealed notable reductions in the elevated number of calcified nodules and enhanced ALP activity in AS fibroblasts after silencing miR-92b-3p. Moreover, miR-92b-3p can intrinsically facilitate ALP activity and osteoblast differentiation [28]. Several markers such as RUNX2, OPN, OSX, and COL I have been ascertained as key markers of osteogenesis [32]. Our results elicited vital reductions in the elevated expression levels of RUNX2, OPN, OSX, and COL I in AS fibroblasts after silencing miR-92b-3p. Additionally, miR-92b-3p knockdown has an evident inhibitory function on hypoxia-induced abnormal proliferation of vascular smooth muscle cells [33]. Consistently, silencing miR-92b-3p significantly suppressed AS fibroblast proliferation. Moreover, an existing study has highlighted the ability of miR-92b to facilitate the osteogenic differentiation of mesenchymal stem cells in bone fractures [16]. Collectively, our results demonstrated that silencing miR-92b-3p could extensively suppress the proliferation and osteogenic differentiation of AS fibroblasts.

The vital functionality of TOB1 has been evident in miR-26a-mediated osteogenesis in osteoporosis [21]. Our findings were illustrative of reduced TOB1 expression levels in AS fibroblasts relative to those in normal fibroblasts. The binding site of miR-92b-3p and TOB1 was predicted using the ENCORI database, while their target relationship was further verified. Additionally, our results revealed that the decreased TOB1 expression in AS fibroblasts was elevated after silencing miR-92b-3p. Therefore, the capacity of miR-92b-3p to negatively regulate TOB1 expression is evident. As has been reported, TOB1 downregulation can radically induce ALP activity and OSX expression in rheumatoid arthritis [25]. Our findings demonstrated improved ALP activity along with elevated expression levels of RUNX2, OPN, OSX, and COL I in AS fibroblasts after miR-92b-3p and TOB1 downregulation. TOB1 as a member of the mammalian BTG/TOB family has demonstrated imperative anti-proliferative abilities [34]. Our results highlighted the ability of downregulated TOB1 to fractionally silence miR-92b-3p-mediated inhibition on AS fibroblast proliferation. An existing study has indicated that an inherent TOB1 deficiency can improve new bone formation [35]. Additionally, miR-486-5p induces osteoblast differentiation by decreasing TOB1 expression, while the protein levels of ALP, OSX, AND DLX2 are all increased and the ALP activity and mineralization of osteoblasts are stimulated following downregulation of TOB1 [25]. Conjointly, our findings demonstrated the ability of TOB1 downregulation to marginally annul the inhibitory effect of silencing miR-92b-3p on the proliferation and osteogenic differentiation of AS fibroblasts.

The BMP/Smad pathway has an important function in the osteogenic differentiation of AS fibroblasts [22]. A prior study has documented the ability of TOB1 to negatively regulate the BMP/Smad pathway [36]. Our findings elicited marked downregulation of the increased expression levels of BMP-2 and p-Smad1/5/9/t-Smad1 in AS fibroblasts after silencing miR-92b-3p, while TOB1 downregulation elevated the expression levels of BMP-2 and p-Smad1/5/9/t-Smad1. The activation of the BMP/Smad pathway has been identified frequently during the osteogenic differentiation of stem cells [37]. Our results elicited a reduced number of calcified nodules along with lowered ALP activity and expression levels of RUNX2, OPN, OSX, and COL I after blockade of the BMP/Smad pathway. An existing study has identified a positive correlation between the BMP2/Smad pathway and the proliferation of human periodontal ligament stem cells [38]. Our results exhibited that the proliferative ability of AS fibroblasts was weakened after inhibition of the BMP/Smad pathway. Additionally, miR-486-5p can target TOB1 and activate the BMP/Smad pathway in osteoblasts [25]. A prior study has highlighted the ability of the BMP/Smad pathway to facilitate KR-12-a6-induced osteogenic differentiation of human bone marrow mesenchymal stem cells [39]. Altogether, our findings elicited that miR-92b-3p could successfully manipulate the osteogenic differentiation of AS fibroblasts by inhibition of the TOB1/BMP/Smad pathway.

In conclusion, this study highlighted that miR-92b-3p regulates the osteogenic differentiation of AS fibroblasts by blocking the TOB1/BMP/Smad pathway. However, there are several limitations to our study. The AS fibroblasts used in this study were extracted from 5 patients and cultured for the third generation, whose morphology and immunocytochemistry results showed no difference. Nevertheless, the donor discrepancy may be revealed when the sample size is expanded. Second, the off-target of siRNA by using si-TOB1 was not considered, which shall be further confirmed in future research. Additionally, the regulatory mechanisms of miR-92b-3p in osteogenic differentiation of AS fibroblasts were not thoroughly explored, and the precise regulatory mechanisms of miR-92b-3p and other potential downstream pathways require further elaboration in future studies.

## Supplementary Information

**Additional file 1**. **Supplementary Figure 1:** Protein expression levels of TOB1 determined by Western blot.

**Additional file 2**. **Supplementary Figure 2:** Protein expression levels of TOB1 determined by Western blot.

**Additional file 3**. **Supplementary Figure 3:** Protein expression levels of BMP-2 and p-Smad1/5/9/t-Smad1 determined by Western blot.

## Data Availability

The data that support the findings of this study are available from the corresponding author upon reasonable request.
