# Correction: Functional mechanism of miR-92b-3p in osteogenic differentiation of fibroblasts in patients with ankylosing spondylitis via the TOB1/BMP/Smad pathway

**DOI:** 10.1186/s13018-023-03915-1

**Published:** 2023-06-13

**Authors:** Liansong Lu, Shaohua Sun, Haojie Li, Yingzhi Xie

**Affiliations:** 1 Department of Spinal Surgery, Ningbo No. 6 Hospital, 1059 East Zhongshan Road, Yinzhou District, Ningbo, 315040 Zhejiang China; 2 Department of Medical Image, Ningbo No. 6 Hospital, 1059 East Zhongshan Road, Yinzhou District, Ningbo, 315040 Zhejiang China

**Correction: Journal of Orthopaedic Surgery and Research (2023) 18:402** **https://doi.org/10.1186/s13018-023-03850-1**

Following publication of the original article [1], the authors identified an error in the author name of Haojie Li.

The author group has been updated above and the original article [1] has been corrected.
